# Some Interesting Theories

**Published:** 1920-11-20

**Authors:** 


### SIR JAMES CANTLIE ON INFANT WELFARE.

### Some Interesting Theories.

It is obvious that Sir James Cantlie never anticipates a ready acceptation of his doctrines and precepts: he is, as he puts it, in despair from the beginning, and does not expect to be believed! But he is not out to deal in pleasantries or to reiterate accepted truths, neither would he enjoy an audience totally in agreement with him.

Thirty-five years ago he published his "Degeneration amongst Londoners," which, he affirms, roused such a storm in every newspaper that he hid his head in China. What else could a medical man do when he was caricatured in *Punch*? But when he returned twenty years later and published his reassuring "Physical Efficiency," nobody read it at all. His theory is not unnaturally "Give them something startling and shocking and they will listen; reassure and encourage them, and they will take no interest."

### Country Children and Condensed Milk.

Speaking recently before the National Baby Week Council, Sir James pleaded for the country children. He railed against the tied farm which sells its milk to the townsmen while the children of the very milker must live on condensed stuff. From his own farm he sold only to the people in the neighbourhood, and had started it largely with that intent. Mentioning the daylight-saving scheme, he characterised it as a perversion of nature, and said that it was hated by mothers and farmers, though he knew it was of little use telling this to townsfolk. They would merely say, "He's only an old farmer, and farmers are always grumbling." The town children had better houses, better water, better food; the country children gained alone in better air. Here in London streets there had not been a breath of fresh air for one hundred years, nothing but air that other people had breathed first.

### War Against Perambulators.

"I was not carried about in a perambulator," said Sir James, "I was carried by my nurse or my mother. The temperature of the woman's body is 98.4°, which is what the baby needs. The nurse puts a hot bottle into the perambulator which makes the baby perspire. She forgets—for nurses meet with temptations—to take him home until the bottle is cold, and so the temperature is constantly changing. Do you find prams all over the world? No, you do not. You find them in England and a small strip of Europe, and the Colonies and America, and practically nowhere else at all. If a woman cannot carry her child she is not worthy that God should make her a mother."

### Assaulting a Comforter.

On the temperature of the babies' milk, he said that the mother tested the heat of the bottle by putting it into her mouth. The temperature of the baby's body and the baby's mouth was 98.4°. He had ascertained that a woman could sip tea at 140° from a spoon, at 130° from a cup, and when it was below 120° she considered it cold. So she gave it to the baby at least 22° too hot, it scalded his gums with their fifty-two teeth waiting their turn, and brought the blood to the surface so that it could not feed the tooth.

He then asked the Council if they had succeeded in stopping "comforters"? He would like to make an Act of Parliament whereby it was criminal for a man to sell a comforter. When he saw a baby sucking one he took it away and threw it over the hedge, and then gave the nursemaid sixpence to enable her to prove what had happened! He once travelled with two women each of whom had a baby, each of whom had a comforter. He remonstrated with the women in vain. Just before he alighted, he seized his chance and a comforter with each hand and threw them out of the window. When he watched the train going out he saw the women shaking their fists at him! He advised his audience to do the same—nothing could happen to them, and they could not be taken in charge for merely assaulting a comforter.

